# The efficacy of traditional Chinese medicine exercise therapy for the prevention and treatment of mental health disorders in university students: A protocol for systematic review and network meta-analysis

**DOI:** 10.1097/MD.0000000000028805

**Published:** 2022-02-18

**Authors:** Tuoyu Lu, Zhenhui Lu, Yingzi Yu

**Affiliations:** a Health Science Center Medical Doctor Program, Xi’an Jiaotong University, Xi‘an, Shaanxi Province, China; b Hepatobiliary and Pancreatic Surgery, Shenzhen Qianhai Shekou Free Trade Zone Hospital), Shenzhen, Guangdong Province, China; c Department of Hospital Infection-Control, Shenzhen Qianhai Shekou Free Trade Zone Hospital, Shenzhen, Guangdong Province, China.

**Keywords:** anxiety, depression, network meta-analysis, protocol, traditional Chinese medicine exercise, university students

## Abstract

**Background:**

Mental health disorders are highly prevalent among university students. Mental health is important in the healthy growth and overall development of university students. Many studies have indicated that traditional Chinese medicine (TCM) exercise therapies can alleviate anxiety and depression symptoms in university students. However, their definite efficacy and the optimal choice of TCM exercise therapy remain controversial. In this study, we aim to assess and compare the effects of different TCM exercise therapies on anxiety and depression symptoms in university students by network meta-analysis.

**Methods:**

Randomized controlled trials (RCTs) examining TCM exercise therapies for the anxiety and depression in university students published before January 2022 will be searched in online databases, including the PubMed, Web of Science, Scopus, Cochrane Library, Embase, China Scientific Journal Database, China National Knowledge Infrastructure, Chinese Biomedical Literature Database, and Wanfang Database. Two researchers will be independently responsible for literature screening, data extraction, and assessment of their quality. Standard pairwise and network meta-analysis will be performed to compare the efficacy of different TCM exercise therapies on anxiety and depression symptoms in university students using Stata14.0 and GeMTC0.14.3.

**Results:**

The results of this meta-analysis will be submitted to a peer-reviewed journal for publication.

**Conclusion:**

This meta-analysis will provide the evidence for supporting the intervention strategies of TCM exercise therapy for improving negative emotions such as anxiety and depression among university students.

OSF REGISTRATION NUMBER: DOI 10.17605/OSF.IO/VTGBE.

## 1 Introduction

Mental health is an important aspect of university students’ health, which profoundly affects their healthy growth and comprehensive development.^[^1^–^4^]^ The current psychological health of university students is not optimistic, and a considerable number of university students have psychological problems or bad tendencies.^[^5^,^^6^^]^ If the psychological problems cannot be solved timely and effectively, they may affect the study and life of university students, and even lead to behavioral disorders and serious mental diseases.^[^7^,^^8^^]^ Depression and anxiety are the 2 most prominent psychological problems in university students, both of which are destructive and need to be highly concerned.^[9]^ Serious consequences will be caused if an active management of depression and anxiety in university students is lacked.^[10]^

Traditional Chinese medicine (TCM) exercise therapy belongs to aerobic exercise at a low to moderate intensity, which is popular and easy to be performed.^[11]^ It is widely used to strengthen the body and applied to disease prevention and treatment. There are many types of TCM exercise therapy, mainly including Tai Chi, Wu Qin Xi, Six Healing Sounds, Ba Duan Jin, etc.^[^6^,^^12^^]^ TCM exercise therapy can not only nourish the mind and body of university students, but also calm down the emotions, thoughts, and consciousness, which significantly improves their mental health, emotion management ability, and social adaptation ability, as well as alleviate anxiety and depression.^[13]^

Previous findings have validated the role of TCM exercise therapy in alleviating symptoms of anxiety and depression in university students.^[^13^–^19^]^ However, the optimal choice of TCM exercise therapy remains unclear, because each one has their own advantages and disadvantages. To our knowledge, network meta-analysis (NMA) comparing the efficacy of different TCM exercise therapies on alleviating symptoms of anxiety and depression in university students has not been reported. To promote the rational application of TCM exercise therapy, this study aims to conduct a NMA on randomized controlled trials (RCTs) reporting the application of TCM exercise therapy to alleviate symptoms of anxiety and depression in university students.

## 2 Methods

### 2.1 Study registration

The protocol of this review was registered in OSF (OSF registration number: DOI 10.17605/OSF.IO/VTGBE). This protocol was designed according to the guideline of Preferred Reporting Items for Systematic Review and Meta-Analysis Protocols (PRISMA-P).^[20]^ The findings of this study will be reported in line with the guideline of Preferred Reporting Items for Systematic Reviews and Network Meta-Analysis (PRISMA-NMA).^[21]^

### 2.2 Inclusion criteria for study selection

#### 2.2.1 Types of studies

Eligible RCTs reporting TCM exercise therapies on alleviating anxiety and depression symptoms in university students will be searched and analyzed.

#### 2.2.2 Types of participants

University students at 18 to 26 years with symptoms of anxiety and depression will be included.

#### 2.2.3 Types of interventions

University students with symptoms of anxiety and depression in the treatment group are additionally intervened by TCM exercise therapies, including Tai Chi, Ba Duan Jin, the classics of tendon changing, Six Healing Sounds, and Wu Qin Xi, and those in control group are intervened by conventional health guidance, medication, and care.

#### 2.2.4 Types of outcome indexes

(1) Anxiety assessed by the Self-Rating Anxiety Scale (SAS) and Hamilton Anxiety Scale (HAMA);
(2) Depression assessed by the Hamilton Depression Rating Scale (HAMD) and Self-Rating Depression Scale (SDS).

### 2.3 Exclusion criteria

(1) Repeated literatures;
(2) Non-RCTs, editorials, letters, reviews, etc;
(3) Absence of complete data or full-text.

### 2.4 Data sources

RCTs examining TCM exercise therapies for the anxiety and depression in university students published before January 2022 will be searched in a combination of keywords and subject headings in online databases, including the PubMed, Web of Science, Scopus, Cochrane Library, Embase, China Scientific Journal Database, China National Knowledge Infrastructure, Chinese Biomedical Literature Database, and Wanfang Database. The searching strategy is listed in Table 1.

**Table 1:** Search strategy in PubMed database.

Number	Search terms
#1	University students[Title/Abstract]
#2	College student[Title/Abstract]
#3	undergraduate[Title/Abstract]
#4	OR/1–3
#5	Exercise Therapy[MeSH]
#6	Therapy, Exercise[Title/Abstract]
#7	Exercise Therapies[Title/Abstract]
#8	Therapies, Exercise[Title/Abstract]
#9	Tai Ji[MeSH]
#10	T’ai Chi[Title/Abstract]
#11	Tai Chi[Title/Abstract]
#12	Tai Ji Quan[Title/Abstract]
#13	Tai-ji[Title/Abstract]
#14	Taiji[Title/Abstract]
#15	Taijiquan[Title/Abstract]
#16	Tai Chi Chuan[Title/Abstract]
#17	Chi, Tai[Title/Abstract]
#18	Ji Quan, Tai[Title/Abstract]
#19	Quan, Tai Ji[Title/Abstract]
#20	traditional Chinese medicine exercise therapy[Title/Abstract]
#21	Ba Duan Jin[Title/Abstract]
#22	classics of tendon changing[Title/Abstract]
#23	Six Healing Sounds[Title/Abstract]
#24	Wu Qin Xi[Title/Abstract]
#25	Qi Gong[Title/Abstract]
#26	Liu Zi Jue[Title/Abstract]
#27	OR/5-26
#28	Randomized Controlled Trials as Topic[MeSH]
#29	Clinical Trials, Randomized[Title/Abstract]
#30	Controlled Clinical Trials, Randomized[Title/Abstract]
#31	Trials, Randomized Clinical[Title/Abstract]
#32	Random∗[Title/Abstract]
#33	OR/28-32
#34	#4 AND #27 AND #33

### 2.5 Data collection and analysis

#### 2.5.1 Data extraction and management

**Figure 1:**
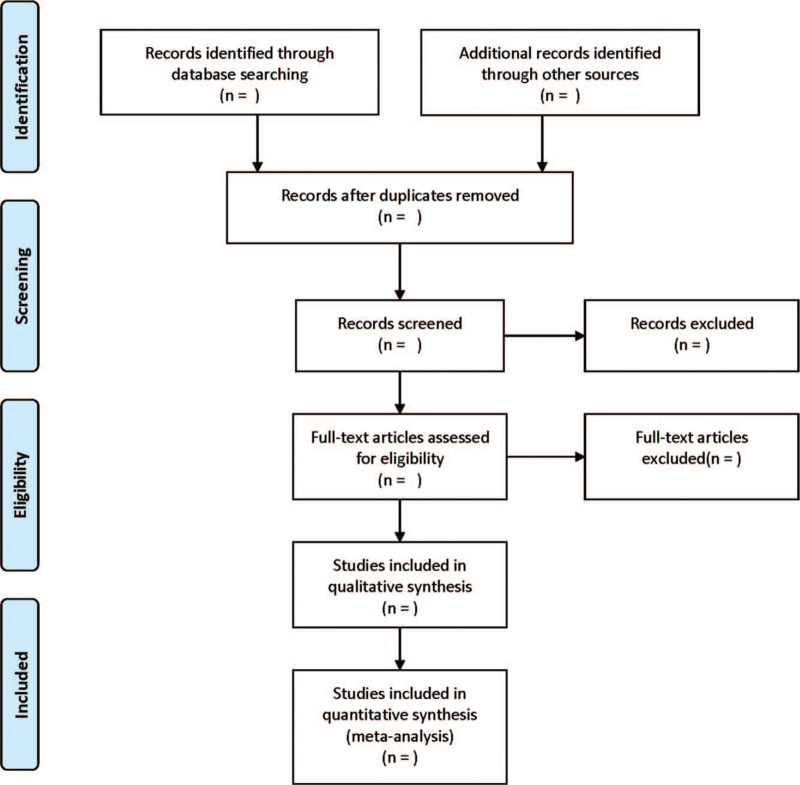
Flow diagram of study selection process.

Two reviewers will be responsible for initial screening of the retrieved literatures, and duplicates will be removed using EndNoteX9. After reviewing full-texts, eligible literatures will be introduced into Excel 2019 for the following data extraction. The following data will be collected: the first author, year, sample size, age, nationality, intervention, length of intervention, and outcome indicators. Any disagreement will be solved by collective discussion of the research team. The flow chart of literature screening is presented in Figure 1.

#### 2.5.2 Assessment of risk of bias

The quality of the literature will be independently assessed by 2 reviewers according to the risk bias assessment criteria established in Cochrane Handbook 5.1.0,^[22]^ and double-checked. Any disagreement will be discussed and resolved collectively by the research team.

#### 2.5.3 Measures of therapeutic effect

The effect size of continuous variable data will be calculated with the standardized mean difference (SMD) and corresponding 95% confidence intervals (CIs).

#### 2.5.4 Management of missing data

In case of any missing data in relevant study, the original data will be requested by e-mail; Otherwise, they will be excluded from this study.

#### 2.5.5 Assessment of heterogeneity and data synthesis

Statistical analysis and graphical plotting will be performed using Stata14.0 (STATA Corporation, College Station, TX) and GeMTC0.14.3. The heterogeneity among the direct comparison results will be assessed by Chi-square test and *I* ^2^ test. If there is no heterogeneity (*I* ^2^ < 50%, *P* > .1), a fixed-effects model will be adopted in the meta-analysis; Otherwise, a random-effects model will be adopted.^[23]^ NMA will be performed via GeMTC0.14.3. Simulations will be performed using 4 chains with 50,000 iterations, involving the first 20,000 used for annealing. Inconsistency between direct and indirect evidence will be tested using nodal splitting and *P* > .05 indicates non-significant inconsistency. The convergence among the included studies will be assessed using the potential scale reduction parameter (PSRF). PSRF close to 1 indicates good convergence and a credible conclusion will be obtained using consistency model analysis. Rank probability ranking plots will be drawn to rank the efficacy of each intervention. Network evidence plots will be drawn by Stata 14.0 for the comparison between treatment group and control group.

#### 2.5.6 Assessment of publication biases

Small sample effects or publication bias of included studies will be assessed by comparison-adjusted funnel plots.^[24]^

#### 2.5.7 Subgroup analysis

Subgroup analysis based on the intervention time will be performed.

#### 2.5.8 Sensitivity analysis

Sensitivity analysis will be performed by a one-by-one elimination method to verify the robustness of the results.

#### 2.5.9 Ethics and dissemination

The contents of this paper do not involve moral approval or ethical review and it will be presented in print or at relevant conferences.

## 3 Discussion

TCM exercise therapy has been applied to enhance physical health in China for thousands of years. Existing systematic evaluations have verified the efficacy of TCM exercise therapy on a variety of chronic diseases.^[^25^,^^26^^]^ Modern university students live and study at a faster pace, who have more stressful interpersonal relationships, and are easily agitated.^[^27^,^^28^^]^ Therefore, TCM exercise therapy is believed to effectively regulate the tension of university students, relieve the psychological tension and pressure, and form a stable psychological state. Long-term exercise also has significant effects on cardiovascular health, flexibility and balance, executive function, self-regulation of the brain and lumbar muscle strength in young healthy people. It remarkably improves physiological and biochemical indicators related to emotions such as 5-hydroxytryptamine, endorphins, and plasma lipocalin. This study will compare the effects of different TCM exercise therapies on anxiety and depression in university students by NMA, thus providing a reference for determining the optimal TCM exercise therapy.

## Author contributions

**Conceptualization:** Tuoyu Lu, Yingzi Yu.

**Data collection:** Tuoyu Lu and Zhenhui Lu.

**Formal analysis:** Tuoyu Lu.

**Funding acquisition:** Yingzi Yu.

**Funding support:** Zhenhui Lu.

**Investigation:** Tuoyu Lu.

**Methodology:** Tuoyu Lu.

**Project administration:** Yingzi Yu.

**Resources:** Tuoyu Lu, Zhenhui Lu.

**Software operating:** Tuoyu Lu and Zhenhui Lu.

**Software:** Zhenhui Lu.

**Supervision:** Yingzi Yu.

**Validation:** Zhenhui Lu.

**Visualization:** Zhenhui Lu.

**Writing – original draft:** Tuoyu Lu and Yingzi Yu.

**Writing – review & editing:** Tuoyu Lu and Yingzi Yu.
